# Inflammation as a mediating pathway between social defeat and mental health in humans: A systematic review

**DOI:** 10.1038/s41398-026-03911-z

**Published:** 2026-04-24

**Authors:** Shivank Sharma, Matthew Taylor, Zara Sadiq, Tanya Kashyap, Hanlei Shao, Zeba Sulaiman, Yiqiao Wang, Balachandran Kumarendran, Sian Lowri Griffiths

**Affiliations:** 1 Institute for Mental Health, University of Birmingham, Birmingham, England; 2 Birmingham Medical School, University of Birmingham, Birmingham, England; 3 Mental Health Research Unit, Sheffield Centre for Health and Related Research, The University of Sheffield, Sheffield, England; 4 Sheffield Health and Social Care NHS Foundation Trust, Sheffield, England; 5 National Institute for Health and Care Research, London, England; 6 Mental Health Mission Midlands Translational Centre, Birmingham, England

**Keywords:** Biomarkers, Scientific community

## Abstract

**Introduction:**

Social Defeat (SD), an animal-derived concept characterised by experiences of subordination, may influence biological mechanisms affecting mental health. Despite evidence linking inflammation to mental disorders and animal studies suggesting SD’s inflammatory effects, to date, no review has comprehensively examined these relationships in human populations. This systematic review synthesises evidence from human studies examining human SD, inflammation, and mental health, with particular attention to inflammation as a potential mediating pathway in these relationships, to better inform understandings of mental health disparities across marginalised communities.

**Methods:**

Embase, MEDLINE, and PsycInfo were searched for eligible observational human studies published from database inception to September 2025. Studies were screened by two reviewers, while a single reviewer extracted data and evaluated study quality and risk of bias. Narrative synthesis revealed overarching themes and patterns.

**Results:**

13 heterogenous studies met the inclusion criteria, predominantly examining social isolation, peer victimisation, discrimination, and childhood adversity. Interleukin-6 (IL-6) and C-Reactive Protein (CRP) with depression were the primarily assessed inflammatory and mental health outcomes. Most studies found SD-inflammation-mental health associations, although some inconsistencies emerged. Sex and SD type arose as potential mediators. However, results were limited by the range of moderate-to-high quality and low-to-high risk of bias across studies.

**Conclusion:**

Various forms of human SD experiences may independently alter immunological profiles and mental health outcomes, providing translational support for animal studies linking SD to inflammatory changes. The mediating role of SD in the relationship between inflammation and mental health remains inconclusive, however. While longitudinal studies may help elucidate the directionality of associations, this study reiterates that SD prevention is paramount for achieving sustainable mental health improvements.

## Introduction

### Mental disorders and social determinants of mental health

Mental disorders such as depression, anxiety and psychosis, are prevalent conditions responsible for substantial morbidity globally [1]. In addition to causing significant mental distress, mental disorders adversely affect the physical health, social functioning, and employment opportunities of those affected by them [1, 2]. The burden of mental disorders upon society has long been recognised to fall unevenly [3], with members of certain groups more likely to be diagnosed with a mental health condition. Marginalised communities, who are typically excluded from economic, social, and cultural participation [4], are disproportionately affected by mental disorders, which further exacerbates the health inequalities experienced by these individuals. Recent work has also highlighted the importance of social stress as a transdiagnostic factor in psychiatric disorders, including bipolar disorder [5].

Despite advancements in psychiatric knowledge, diagnosis, and treatment [6], there remains uncertainty around the precise aetiology of numerous mental disorders [7]. While biological factors play a role, social determinants of mental ill health such as early life adversity, social isolation and discrimination have attracted increasing attention [8]. Elucidating the biological mechanisms potentially mediating these relationships between social determinants and disparate mental health outcomes may help in developing effective early intervention and prevention strategies [9], improving public health measures [10], and reducing mental health disparities [11].

### Inflammation as a potential mediating pathway between social defeat and mental health

One theory that considers these aetiological uncertainties concerns the role of immune dysregulation and inflammation in modulating neurogenesis, neurotransmission, and neuroplasticity in the pathogenesis of mental disorders [12, 13]. Inflammation is an immune-mediated response, serving as a protective mechanism against tissue injury and infection [14]. While the adaptive immune response provides a tailored defence against specific threats, the innate response triggers a rapid cytokine release that includes both pro- (e.g., IL-1β, IL-6, TNF-α) and anti-inflammatory (e.g., IL-10, TGF-β) mediators regulating immune balance and neural signalling [15, 16]. In the central nervous system (CNS), cytokines arise from microglia, astrocytes, neurons, and endothelial cells, all shaping neuroimmune signalling; chronic elevation can disrupt neurotransmitter synthesis and reuptake [17]. In addition to resident innate immune cells, evidence from neuroimmunology indicates that adaptive immune cells — including T and B lymphocytes — are also present within the healthy CNS, where they contribute to immune surveillance and maintenance of neural homeostasis [17, 18]. Such neurotoxic effects are exacerbated by increased blood-brain barrier (BBB) permeability during inflammation, potentially allowing peripheral inflammatory cytokines and immune cells to enter the CNS, driving neuroinflammation [16, 18].

The role of inflammation in mental disorders involves complex interactions between the immune system, brain function, and environmental stressors. Proinflammatory cytokines such as interleukin-1β (IL-1β), interleukin-6 (IL-6), and tumour necrosis factor-α (TNF-α) can influence neurotransmission and neuroplasticity, contributing to symptoms across depression, anxiety, and psychosis in humans [19–21]. Cytokines such as IL-6 and TNF-α may alter the kynurenine pathway and downstream neurotransmission, though evidence remains correlational rather than causal [22, 23]. Elevated C-reactive protein (CRP) and evidence of possible alterations in blood–brain-barrier (BBB) integrity have been reported in some psychiatric conditions, suggesting—though not confirming—a link between peripheral immune activation and central processes [24–27]. While such mechanisms illustrate plausible biological routes, the specific role of social experiences such as social defeat (SD) in modulating inflammatory responses remains inconclusive. Findings from rodent models demonstrate that SD induces immune activation and dopaminergic sensitisation, but human research remains limited and fragmented [28–36]. The present review therefore seeks to integrate human studies examining associations between SD, inflammation, and mental-health outcomes, to clarify shared mechanisms and identify priorities for future research. SD can be viewed as a psychosocial stressor that interacts with immune pathways contributing to mental disorders. Rather than an alternative hypothesis, SD complements immune-based models by linking social experience to biological change [37, 38].

### Conceptualisation and evolution of the social defeat model

Historical conceptualisations of SD have typically utilised animal models [39], examining the social hierarchies arising between dominant and subordinate rats. The subordinate rat is said to experience SD following the confrontation that ensues when it is placed into the territory of a dominant rat [40], which can model acute or chronic SD depending on the duration of exposure. SD as a model of social stress opened several avenues for early animal research into the biomolecular consequences of stress, demonstrating stress-mediated impairments of corticosterone [41], dopaminergic [42], and immunological responses and function [43]. More recently, animal literature has identified several downstream biological changes potentiated by SD, including increased dopamine function [28] as well as elevated pituitary adenylate cyclase-activating polypeptide (PACAP) and PACAP type 1 receptor activation within the amygdala [29].

Of relevance to the immune dysregulation hypothesis of mental disorders is the association of SD with peripheral inflammation [30] and the mediation of neuroinflammation by SD secondary to microglial activation within the CNS [31, 32]. Animal studies indicate that exposure to SD can elevate proinflammatory cytokines IL-1 and IL-6, which may contribute to the development of social avoidance behaviours [33–36]. These findings have been applied to the vulnerability-stress-inflammation model for schizophrenia, suggesting that genetic predispositions and early life experiences contribute to a pro-inflammatory state which disrupts neurotransmission and alters brain structure [23, 44]. Within this model, inflammation consequently serves as a plausible biological pathway by which real-world SD, such as that experienced by marginalised populations, contributes to adverse mental health outcomes.

### From animal models to human applications of social defeat

Beyond this, chronic SD could be the link between the increased risk of schizophrenia observed among migrants, a hypothesis seemingly potentiated by animal-established models of SD-mediated sensitisation of the mesolimbic dopamine system [45]. While this research focuses on ethnicity-related SD, the concept’s scope has been expanded to encompass discrimination, childhood bullying, and sexual minority status [40], outlining SD as a real-world phenomenon that captures marginalisation perpetrated by dominant social agents. Consequently, human applications of SD are diverse and may incorporate elements of bullying, racism, childhood trauma, social stress, and isolation - experiences that reflect perceptions of subordination, exclusion, or marginalisation within social hierarchies and increase individual vulnerability to mental illness [46–50]. These experiences were conceptualised as distinct but convergent manifestations of SD, each reflecting perceptions of subordination, exclusion, or marginalisation within social hierarchies.

### The social signal transduction framework and rationale for this review

One relevant theory of human-focused SD is the social transduction framework, which posits that social stress can upregulate inflammatory activity and contribute to the onset and exacerbation of depressive symptoms [51], with individuals who exhibit stronger inflammatory responses to social stress being at an elevated risk of developing depression [52]. Furthermore, inflammatory reactivity may be attributed to the positive relationship between repeated interpersonal stress and depressive symptoms [53]. Similarly, adverse childhood experiences (ACEs) in the context of bullying have been associated with promoting inflammation through neural, endocrine, immune, and metabolic inflammatory pathway mediation [54]. Concurrently, bullying has been associated with anxiety, depression, and self-harm [55]. Similarly, repeated racial discrimination may mediate chronic activation of the sympathetic nervous system and the hypothalamic-pituitary-adrenal (HPA) axis, potentially modulating inflammation-related genes via catecholamines and cortisol [56]. In fact, meta-analytic data in children and youth indicate that racism is associated with higher CRP and IL-6 [57] underscoring discrimination as a plausible real-world SD exposure with inflammatory correlates. Simultaneously, racial discrimination is associated with the development of psychotic and substance use disorders [58].

While animal studies have identified associations between SD and inflammation [33–36], human research in this area remains limited. As the causal mechanisms underlying numerous mental disorders remain inconclusive [7], there is a need for further research identifying potentially relevant aetiological factors contributing to their development. Consequently, considering the outlined animal-based evidence linking SD with the immune dysregulation hypothesis of mental disorders, it is plausible that SD mediates inflammatory pathways in humans that contribute to the development of mental disorders.

Given the multifaceted nature of social-defeat experiences and their associations with several psychiatric outcomes, this review adopted a broad trans-diagnostic perspective—encompassing depression, PTSD, internalising/externalising difficulties, and loneliness—to identify potential shared inflammatory mechanisms underlying these conditions.

To date, no comprehensive review has synthesised the diverse elements of human SD, inflammation, and associated mental health outcomes. This review sought to expand beyond animal-based definitions of SD to include human experiences of marginalisation including bullying, racism, and social exclusion, and explore the association of these with inflammatory processes and diverse mental health outcomes via narrative synthesis. Finally, this review aimed to identify sources of heterogeneity and limitations in current evidence to guide future research directions and formulate recommendations for advancing human SD research in the context of mental health. Although the concept of SD was originally developed in relation to psychosis and schizophrenia, the eligible human studies primarily examined non-psychotic outcomes; therefore, this review focuses on these broader mental-health associations. A glossary of key terms used throughout this review is provided following the abstract and key words for reader reference.

## Methods

### Study design

Following best practices in systematic review methodology [59], this study adhered to PRISMA guidelines [60] ensuring transparent standardised reporting of the review process and findings (see Supplementary Tables S.1–S.3). This review was not registered.

### Eligibility criteria

This systematic review included empirical experimental or observational studies of populations with human experiences of SD (e.g. experiences of marginalisation including bullying, racism, and social exclusion), markers of inflammation and mental health outcomes, published in English across all ages, sexes, races, and ethnicities from database inception. Comparatively, animal studies, articles such as posters, conference abstracts, book chapters, commentaries, systematic reviews, editorials, meta-analyses, unpublished studies, and opinion pieces were excluded.

### Operational definitions

For consistency and transparency, key constructs were operationally defined prior to data extraction. The exposure *social defeat* was defined as human experiences of perceived or actual subordination, exclusion, or marginalisation by dominant social groups, encompassing constructs such as bullying, discrimination, social isolation, or early-life adversity. The mediator *inflammatory markers* were defined as circulating cytokines or immune proteins measured in peripheral blood, including IL-6, CRP, TNF-α, fibrinogen, NF-κB, and related transcriptional or regulatory markers. *Mental-health outcomes* referred to quantitative indicators of psychological symptoms or diagnoses—such as depression, anxiety, PTSD, internalising/externalising difficulties, or loneliness—assessed using validated measurement tools. These definitions guided study inclusion and synthesis. The specific operationalisation of SD used in each included study was recorded during data extraction and is presented in Table 1.

**Table 1:** Summary of Included Studies on Social Defeat, Inflammatory Markers, and Mental-Health Outcomes.

**Study (Ref ID; Country)**	[78]; Germany
**Design / Sample Characteristics**	Cross-sectional; n = 1,547 (25–74 yrs); Male 54.75%, Female 45.25%; mean age 50.38; Quality: Moderate; Risk of bias: Moderate
**Social Defeat Construct (Measure)**	Social isolation (Social Network Index)
**Inflammatory Marker(s) and Assay Method**	Serum IL-6 (Sandwich ELISA); CRP (High sensitivity immunoradiometric assay)
**Mental-Health Outcome (Measure)**	Depressive Symptomology (Depression and Exhaustion Subscale) (commonly assessed using CES-D, PHQ-9, BDI-II, or SMFQ).
**Analytical Approach**	Cross-sectional regression
**Key Findings / Direction of Association**	Sex-specific effects: isolation and depressed mood associated with ↑ IL-6 in men; no isolation–CRP association overall; isolation × depressed mood → synergistic ↑ CRP & IL-6.

**Study (Ref ID; Country)**	[74]; Japan
**Design / Sample Characteristics**	Cross-sectional; n = 624 (18–92 yrs); Male 47%, Female 53%; mean age 51.4; Quality: High; Risk: High
**Social Defeat Construct (Measure)**	Social isolation and loneliness (Social Network Index)
**Inflammatory Marker(s) and Assay Method**	Serum neutrophils & lymphocytes (Sysmex XN-1000); Serum CRP (Behring Nephelometer II, nephelometry)
**Mental-Health Outcome (Measure)**	Loneliness (Japanese 10-item UCLA v3)
**Analytical Approach**	Cross-sectional regression
**Key Findings / Direction of Association**	Men: isolation + loneliness → ↑ NLR (chronic inflammation). Women: loneliness correlated with lower CRP; depressive scores did not differ between isolated-lonely vs isolated-non-lonely.

**Study (Ref ID; Country)**	[75]; Taiwan
**Design / Sample Characteristics**	Retrospective cohort; n = 757 ( > 50 yrs); Male 56.3%, Female 43.7%; Quality: Moderate; Risk: Moderate
**Social Defeat Construct (Measure)**	Social isolation (self-created composite score 0–4)
**Inflammatory Marker(s) and Assay Method**	WBC count; % neutrophils, % lymphocytes; NLR; IL-6 (assays not specified)
**Mental-Health Outcome (Measure)**	Depression (CES-D); Loneliness (single item within CES-D)
**Analytical Approach**	Longitudinal/retrospective regression
**Key Findings / Direction of Association**	Isolation associated with depressive symptoms, but attenuated after adjustment; isolation did not increase loneliness in men, doubled risk in women; isolated men showed slightly ↑ IL-6; biomarkers similar by isolation status.

**Study (Ref ID; Country)**	[83]; United States of America
**Design / Sample Characteristics**	Cross-sectional; n = 728; Male 94.51%, Female 5.49%; mean age 58.5; Quality: High; Risk: Moderate
**Social Defeat Construct (Measure)**	Social isolation (Social Network Index); Perceived social support (MSPSS)
**Inflammatory Marker(s) and Assay Method**	hs-CRP (BNII nephelometer); WBC (Beckman Coulter LH 750); Fibrinogen (Clauss assay)
**Mental-Health Outcome (Measure)**	PTSD symptoms (CAPS) (typically measured with instruments such as CAPS or PCL-C).
**Analytical Approach**	Cross-sectional regression
**Key Findings / Direction of Association**	No overall isolation–inflammation link; PTSD status moderated association: isolated non-PTSD → ↑ hs-CRP; PTSD group showed no change.

**Study (Ref ID; Country)**	[72]; United Kingdom
**Design / Sample Characteristics**	Longitudinal; n = 4,583 (7–11 yrs); Male 51.1%, Female 48.9%; Quality: High; Risk: Moderate
**Social Defeat Construct (Measure)**	Peer victimisation (Child-reported Bullying & Friendship Interview Schedule); Stressful life events (mother checklist) (self-reported bullying or peer-victimisation scales).
**Inflammatory Marker(s) and Assay Method**	Serum IL-6 (ELISA); CRP (automated particle-enhanced immunoturbidimetric assay)
**Mental-Health Outcome (Measure)**	SDQ Internalising/Externalising (mother-completed)
**Analytical Approach**	Longitudinal/retrospective regression
**Key Findings / Direction of Association**	Peer victimisation → ↑ IL-6 and ↑ CRP; IL-6 partially mediated effects on peer problems; no mediation for stressful life events.

**Study (Ref ID; Country)**	[76]; United States of America
**Design / Sample Characteristics**	Cross-sectional; n = 91; Male 46.2%, Female 53.8%; mean age 15.85; Quality: High; Risk: High
**Social Defeat Construct (Measure)**	Social & physical peer victimisation (PAA of Child Self-Experiences Questionnaire + Direct and Indirect Aggression Scale)
**Inflammatory Marker(s) and Assay Method**	Serum CRP and IL-6 (ELISA)
**Mental-Health Outcome (Measure)**	CES-DC (Depressive symptoms)
**Analytical Approach**	Cross-sectional regression
**Key Findings / Direction of Association**	Social victimisation → ↑ depressive symptoms, ↑ IL-6 & ↑ CRP; adjusting for waist-to-height removed IL-6 association but not CRP; physical victimisation → ↓ IL-6 & ↓ CRP; not linked to depressive symptoms; no significant mediation.

**Study (Ref ID; Country)**	[71]; Switzerland
**Design / Sample Characteristics**	Cross-sectional; n = 31 (7–16 yrs); Male 33.33%, Female 66.66%; mean age 10.6; Quality: Moderate; Risk: Moderate
**Social Defeat Construct (Measure)**	Childhood maltreatment (child protection team determination)
**Inflammatory Marker(s) and Assay Method**	Lymphocytes, B cells, NK cells, monocytes, total T cells (FACS); CD3 + T-cell subsets incl. T-helper, naïve, cytotoxic, RTE; activation via HLA-DR
**Mental-Health Outcome (Measure)**	PTSD (German PTSD Reaction Index); Depression severity (Child Depression Inventory) (commonly assessed using CES-D, PHQ-9, BDI-II, or SMFQ).
**Analytical Approach**	Cross-sectional regression
**Key Findings / Direction of Association**	In maltreated youth: PTSD → ↓ RTE cells; depression → ↓ RTE and ↓ T-helper cells. Maltreatment alone → ↑ lymphocytes & ↑ T cells; effects diminished when depression co-occurred ( ↓ RTEs).

**Study (Ref ID; Country)**	[73]; United Kingdom
**Design / Sample Characteristics**	Retrospective cohort; n = 3,931 (0–23 yrs); Male 40%, Female 60%; Quality: Moderate; Risk: Low
**Social Defeat Construct (Measure)**	Adverse Childhood Experiences (parent- or child-reported across domains)
**Inflammatory Marker(s) and Assay Method**	Serum CRP (automated particle-enhanced immunoturbidimetric assay); IL-6 (assay not reported)
**Mental-Health Outcome (Measure)**	Short Mood and Feelings Questionnaire (Depressive symptoms) (commonly assessed using CES-D, PHQ-9, BDI-II, or SMFQ).
**Analytical Approach**	Longitudinal/retrospective regression
**Key Findings / Direction of Association**	ACEs 0–7 yrs: no CRP association but ↑ depression risk. Sexual abuse 12–18 yrs: ↑ CRP in men. Bullying 7–12 yrs and household dysfunction 12–18 yrs: ↑ CRP and ↑ depression risk. CRP mediation null/non-significant.

**Study (Ref ID; Country)**	[79]; United States of America
**Design / Sample Characteristics**	Cross-sectional; n = 300 (36–85 yrs); Male 35%, Female 65%; mean age 53.90; Quality: High; Risk: High
**Social Defeat Construct (Measure)**	Lifetime & daily discrimination (self-created summary index) (often measured with Schedule of Racist Events or Perceived Ethnic Discrimination Questionnaire).
**Inflammatory Marker(s) and Assay Method**	IL-6, CRP, E-selectin, ICAM-1, fibrinogen (composite inflammation burden; assays not specified)
**Mental-Health Outcome (Measure)**	Mood and Anxiety Questionnaire: Depression (General Distress subscale) and Anxiety (Anxious Arousal subscales) (commonly assessed using CES-D, PHQ-9, BDI-II, or SMFQ).
**Analytical Approach**	Cross-sectional regression
**Key Findings / Direction of Association**	Greater lifetime discrimination → ↑ inflammatory burden; correlations with anxiety/depression were minor and non-significant.

**Study (Ref ID; Country)**	[77]; United States of America
**Design / Sample Characteristics**	Retrospective cohort; n = 160 (17–22 yrs); Quality: Moderate; Risk: Low
**Social Defeat Construct (Measure)**	Racial discrimination (Schedule of Racist Events questionnaire)
**Inflammatory Marker(s) and Assay Method**	IL-1β, IL-6, IL-8, IL-10, TNF-α, IFN-γ (electrochemiluminescence, SECTOR Imager 2400 A)
**Mental-Health Outcome (Measure)**	CES-D (Depressive symptoms)
**Analytical Approach**	Longitudinal/retrospective regression
**Key Findings / Direction of Association**	Discrimination → ↑ inflammatory markers and ↑ depressive symptoms; inflammation–depression correlation non-significant.

**Study (Ref ID; Country)**	[82]; Canada
**Design / Sample Characteristics**	Prospective longitudinal; n = 103; Female = 100%; mean age 17.19; Quality: Moderate; Risk: Moderate
**Social Defeat Construct (Measure)**	Chronic interpersonal stress (UCLA Life Stress Interview index)
**Inflammatory Marker(s) and Assay Method**	mRNA for NF-κB, GR-β, IκB (qPCR); Serum CRP (high-sensitivity chemiluminescence); IL-6 (high-sensitivity ELISA); Leukocyte IL-6 after LPS stimulation
**Mental-Health Outcome (Measure)**	Beck Depression Inventory
**Analytical Approach**	Longitudinal/retrospective regression
**Key Findings / Direction of Association**	Chronic stress unrelated to baseline CRP/IL-6; stress predicted ↑ IL-6 after LPS stimulation; ↑ NF-κB, GR-β, IκB mRNA; IκB mRNA positively correlated with depression.

**Study (Ref ID; Country)**	[80]; Brazil
**Design / Sample Characteristics**	Longitudinal cohort (2 waves); n = 298 youth; mixed sex; Quality: Moderate; Risk of bias: Moderate.
**Social Defeat Construct (Measure)**	Childhood adversity & life stress composites (child maltreatment; stressful life events; threat; deprivation) — questionnaire composites within BHRCS.
**Inflammatory Marker(s) and Assay Method**	Transcriptional signature: targeted RNA-seq of 78 immune/stress genes in whole blood; analysis focused on differential expression (DESeq2).
**Mental-Health Outcome (Measure)**	Psychopathology (Child Behavior Checklist): p-factor; internalising; externalising.
**Analytical Approach**	Linear mixed models across two timepoints; covariate adjustment; exploratory subgroup analyses.
**Key Findings / Direction of Association**	Internalising symptoms associated with NR3C1 (↓), HSPBP1 (↓), SIN3A (↓), SMAD4 (↓), CRLF3 (↓) and FAR1 (↑); externalising associated with USP38 (↓). No mediation or moderation of adversity → symptoms via gene expression detected. Supports links between adversity and immune-related gene expression but no evidence that transcriptional changes mediate SD → MH pathways in this cohort.

**Study (Ref ID; Country)**	[81]; USA
**Design / Sample Characteristics**	Experimental fMRI with repeated blood sampling; adolescent females, n = 52 (~15 y); 42% with maternal depression history; Quality: Moderate; Risk of bias: Moderate.
**Social Defeat Construct (Measure)**	Chronic interpersonal/social stress vulnerability operationalised via neural threat processing during a negative social evaluation task (social-evaluative stress paradigm).
**Inflammatory Marker(s) and Assay Method**	Transcriptional signature: time-course whole-blood gene expression (baseline, +35, +65 min); pathway-level shifts in innate immune programs (e.g., HIF-1, interferon signaling).
**Mental-Health Outcome (Measure)**	Depressive symptoms / general mental health & wellbeing indices (self-report).
**Analytical Approach**	Brain activity/connectivity → Δgene expression associations; moderation by maternal depression history.
**Key Findings / Direction of Association**	Negative social evaluation elicited robust up-/down-regulation of innate immune pathways. ACC–insula and insula–vmPFC connectivity predicted greater/lesser inflammatory transcriptional shifts, respectively; effects moderated by maternal depression history. Demonstrates neuroimmune coupling to social threat in adolescents—consistent with SD-linked immune activation.

Each study is presented as a separate block summarising its design, sample characteristics, social-defeat construct, inflammatory markers, analytical approach, key findings, and quality assessment. Measurement instruments for childhood maltreatment, depressive symptomatology, and other psychological outcomes are detailed in the relevant rows, corresponding to the operational definitions provided in the Methods.

Inclusion criteria encompassed observational human studies reporting quantitative associations between experiences of SD, inflammatory markers, and mental-health outcomes. Exclusion criteria comprised animal studies, interventional or experimental trials, qualitative research, and studies involving physical, neurodevelopmental, or neurodegenerative comorbidities. Given the large number of initially identified papers, the inclusion and exclusion criteria were iteratively refined prior to full-text screening to improve specificity while maintaining alignment with the review’s objectives. These refinements primarily involved restricting inclusion to observational human studies, excluding qualitative and interventional designs, and clarifying operational definitions of social-defeat constructs to improve conceptual consistency. This process was undertaken in accordance with Cochrane Handbook (v6.4) guidance to ensure methodological transparency and reproducibility [61]. These inclusion and exclusion procedures were developed and refined in accordance with PRISMA 2020 and Cochrane Handbook (v6.4) guidance to ensure methodological transparency and reproducibility.

### Search strategy

Systematic searches of titles and abstracts were conducted across Embase, MedLine, PsycInfo, and Web of Science databases. Boolean operators were used to search the literature by combining key synonyms of select terms with ‘OR’ alongside the following overarching structure: Social Defeat AND Inflammation AND Mental health. No date limits were applied; all databases were searched from inception through May 2024 to ensure comprehensive coverage of the literature. Initial searches using Web of Science returned an overwhelming number of studies (>40,000). Upon further evaluation of these results, many did not appear to be relevant to this review’s research question. This likely resulted from inclusion of the search term ‘il’ which, while intending to identify studies investigating interleukins, captured unrelated abstracts. A reproducible record of the comprehensive search strategy employed in this review can be found in Supplementary Table S.1 per PRISMA-S search strategy reporting guidelines [62].

### Search update and eligibility

While writing the manuscript, the search was updated to capture records from May 2024 through September 2025 (inclusive), including *online-first/ahead-of-print* publications and preprints in indexed servers (e.g., medRxiv/bioRxiv). Conference abstracts were screened but, unless they reported analysable biomarker–mental health results, they were summarised narratively rather than included in the main synthesis.

Newly identified studies were eligible if they (i) operationalised human social-defeat–relevant exposure (e.g., bullying/peer victimisation, discrimination/racism, social exclusion or isolation, threat/deprivation forms of adversity, or experimentally induced negative social evaluation), (ii) measured inflammatory or immune markers (circulating proteins or immune-related gene expression), and (iii) reported quantitative mental-health outcomes (diagnoses or validated symptom scales).

### Study selection

**Fig. 1:**
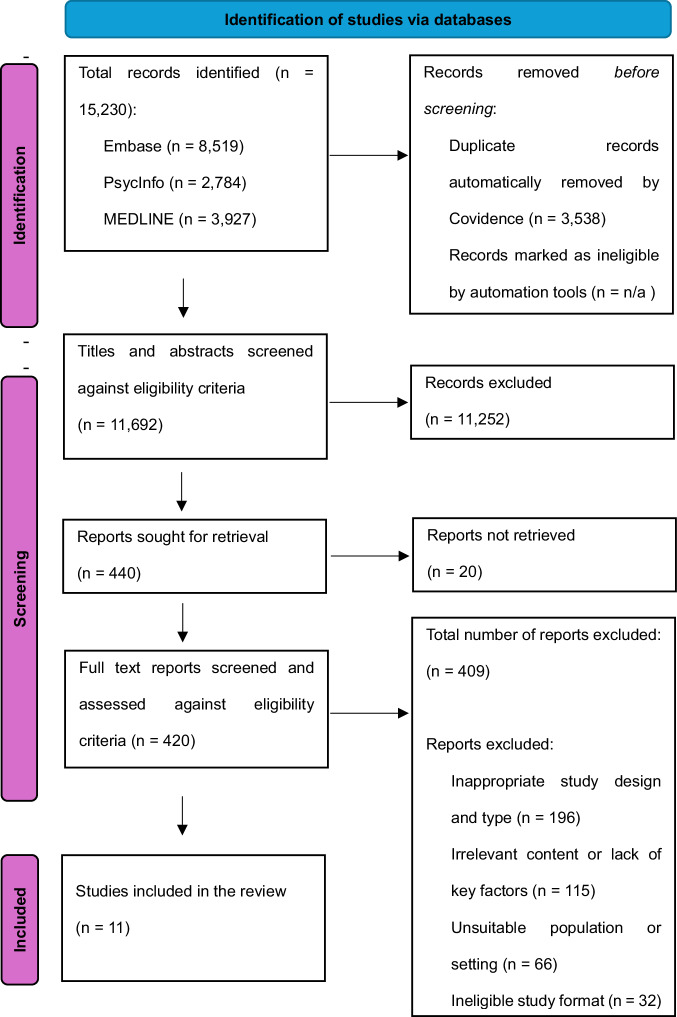
PRISMA Flow Diagram for Databases and Registers (Initial Search). The flow chart details the databases searched up to May 2024, and the removal of non-relevant articles against our eligibility criteria. The subheadings describe the process of this screening, while arrows detail at which point studies were removed or proceeded to the next stage of screening and or anlysis for this review.

Covidence (Veritas Health Innovation, 2020) open-access automation software was used to streamline the study selection and screening process of this review. Studies identified from the initial literature search were uploaded onto Covidence. Automated deduplication by Covidence resulted in the removal of 3538 articles (Fig. 1 [PRISMA]). Two independent reviewers, randomly selected from a six-person screening team (SS, ZS, TK, HS, ZS, YW) via Covidence’s automated allocation system [63], systematically screened each title and abstract. During instances of disagreement, a panel of two reviewers was convened to resolve any conflicts and ensure consistency in the selection process. Of these remaining studies, the same process was conducted to screen full texts with disagreements resolved by a panel of two reviewers, ensuring consistency with the eligibility criteria. This resulted in a final set of papers eligible for this review.

### Quality assessment and risk of bias tools

Eligible studies were quality assessed via the Quality Assessment with Diverse Studies (QuADS) tool due to its ease of use, reliability, and demonstrated content validity while appraising across various study designs [64]. One reviewer (SS) independently utilised the QuADS tool, scoring each of the 13 domains from 0–3. Scores were subsequently summed and compared against a reviewer-formulated QuADS scoring system, providing a descriptive marker of overall study quality.

The use of QuADS was intended to ensure consistency across studies with diverse observational designs (cross-sectional and longitudinal) while capturing broader dimensions of methodological and reporting quality, such as conceptual clarity, data integrity, and reflexivity. We recognise that QuADS does not provide the detailed internal-validity and confounding assessment afforded by quantitative-specific tools such as the Newcastle–Ottawa Scale (NOS) or ROBINS-I. To mitigate this limitation, we complemented QuADS with the Quality Assessment Tool for Quantitative Studies (QATQS), which enabled more targeted evaluation of bias domains and overall risk of bias across the included quantitative studies.

Following this, studies were evaluated for their ROB using the reliable and valid Quality Assessment Tool for Quantitative Studies (QATQS) instrument [65, 66] due to its versatility in appraising several study designs, within which domains are rated as strong, moderate, or weak. Each study’s ROB was determined by a single reviewer (SS) by examining the number of weak domains and assigning a bias risk level. Studies rated weak were considered to have a high ROB. To enhance reliability, a second reviewer (BK) cross-checked a random subset of assessments, and any discrepancies were discussed and resolved by consensus in accordance with tool guidance.

### Study outcomes and data extraction

The primary outcome measurements for this review were types of human social-defeat SD experiences, inflammatory markers, and mental health outcomes. Additional information on study characteristics, participant characteristics (age, sex, ethnicity), and study design were extracted to provide contextual understanding.

Data were extracted using a standardised form developed in Microsoft Excel to ensure consistency, following Cochrane Handbook (v6.4) guidance [67]. Extracted variables included study identifiers (author, year, country, reference ID), study design, sample size and demographics, operationalisation of SD constructs and measurement tools, inflammatory markers and assay methods, mental-health outcomes and psychometric instruments, key findings (direction and statistical significance of associations), and quality-assessment ratings with risk-of-bias classification. The extracted information is summarised in Table 1, organised by SD type, inflammatory marker, and mental-health outcome.

### Data synthesis

Studies reporting relationships between SD, inflammatory markers, and mental health outcomes in human populations were eligible for synthesis and validated against predefined eligibility criteria. Given the heterogeneity of study designs, exposures, and outcomes, a quantitative meta-analysis was not feasible. Instead, findings were summarised narratively following the framework proposed by Popay et al. [68], which provides structured guidance for developing a preliminary synthesis, exploring relationships within and between studies, and assessing the robustness of findings. Studies were organised by the main forms of social-defeat experience (social isolation, peer victimisation, early-life adversity or childhood maltreatment, and discrimination or chronic stress) and examined with respect to their reported inflammatory markers, mental-health outcomes, and potential moderators such as age and sex, in accordance with PRISMA 2020 recommendations. Heterogeneity was explored descriptively by organising studies into analytical subgroups based on the type of social-defeat experience and participant sex, and by comparing patterns of association across study designs, populations, and biomarker types. Quality-assessment results (QuADS and QATQS) were used to inform the narrative synthesis, with greater interpretive emphasis placed on studies rated as strong or moderate quality and low risk of bias.

### Rationale for narrative synthesis

A narrative synthesis [69] was conducted to permit comparison of findings across studies, and to explore key patterns and relationships between SD, inflammatory markers, and mental health outcomes. This approach was selected as the most appropriate method for data synthesis given the heterogeneity in study design, operationalisations of SD, inflammatory markers, and outcome measures across included studies [70]. The synthesis followed the framework proposed by Popay et al. [68], which provides structured guidance for developing a preliminary synthesis, exploring relationships within and between studies, and assessing the robustness of findings.

Utilising data from the completed data extraction form, studies were organised according to the main forms of SD (e.g., social isolation, peer victimisation, early-life adversity, and discrimination or chronic stress) and compared across inflammatory and mental-health outcomes. Where possible, the synthesis explored moderating factors such as age and sex through descriptive observations and subgroup comparisons. Finally, a review of theorised mechanisms linking SD, immune dysregulation, and mental health outcomes was undertaken in accordance with PRISMA 2020 recommendations.

### Conduct of heterogeneity and sensitivity investigations

Due to the descriptive nature of this review, formal statistical tests for heterogeneity or sensitivity analyses are not possible. To address this limitation, preliminary descriptive group comparisons and narrative discussions were conducted to examine heterogeneity and potential influencing factors. Potential moderators were pre-defined a priori as age and sex, given established evidence that both factors influence inflammatory responses and psychosocial stress reactivity [17, 19]. Additional exploratory comparisons considered differences in SD constructs (e.g., social isolation, discrimination, early-life adversity) and inflammatory markers examined. While not a statistical technique, this approach provides insights into possible group differences and trends in the data, offering a preliminary understanding of how various study features might influence outcomes.

## Results

### Literature search and screening process

The initial literature search yielded 15,230 results (Fig. 1*[PRISMA]*). Following automated deduplication via Covidence [63], 11,692 studies remained. 440 studies remained after title and abstract screening, of which 420 were retrieved for full-text screening.

Among our search results was a substantial proportion of experimental studies investigating acute laboratory-generated stress, rather than naturalistic experiences of SD. Further, many studies involved comorbid populations, potentially introducing confounding influences on inflammation. In line with Cochrane guidance [61], the unexpectedly large volume of papers, coupled with the practical constraints of this review, necessitated iterative refinement of the exclusion criteria prior to full-text screening. Resultantly, experimental investigations and studies involving participants with physical, neurodevelopmental, and neurodegenerative comorbidities were additionally excluded. Notably, neuropsychiatric comorbidities were also excluded with the intention of removing neurological conditions with psychiatric manifestations, such as Alzheimer’s dementia, while retaining primary mental health conditions. Full-text screening, following these refined exclusion criteria, yielded 11 eligible studies.

**Fig. 2:**
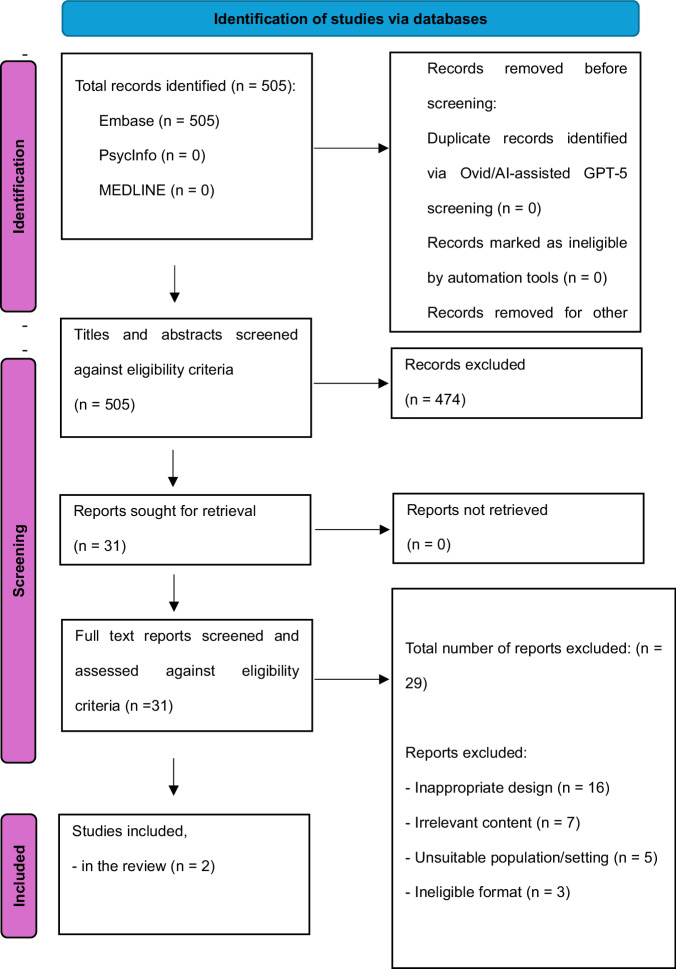
PRISMA Flow Diagram for Databases and Registers (Updated Search). The flow chart details an updated search between May 2024 to September 2025 of the same databases used in the first search, where non-relevant articles were removed from the review as compared to this study's eligibility criteria. The subheadings describe the process of this screening, while arrows detail the points at which studies were removed or progressed through the screening process to reach the next step of screening and or analysis.

An updated search between May 2024 to September 2025 identified 505 articles, of which two additional human studies met the inclusion criteria (Fig. 2*[PRISMA]*). As a result, 13 studies were eligible for narrative synthesis.

### General study characteristics

The 13 included studies, published between 2009 and 2025, explored the relationships between human SD experiences, inflammation, and mental health outcomes. These studies employed both cross-sectional (n = 6) and longitudinal cohort designs (n = 7). Sample sizes greatly varied, ranging from 31 [71] to 4,538 [72], with a median of 300 participants. Research was primarily conducted in Europe and North America, with notable concentrations in the United States (n = 5) and the United Kingdom (n = 2). The studies covered a broad age range from the prenatal period [73] up to 92 years [74], with most focusing on individuals ≤25 years (n = 8). Sex distribution varied considerably across studies, as did ethnic representation, though some studies lacked comprehensive reporting on ethnicity.

### Quality assessment and risk of bias

While these studies were rated as either moderate (n = 8) or high (n = 5) quality, risk of bias varied through low (n = 2), moderate (n = 8), and high (n = 3). Notably, all high-risk studies were cross-sectional.

### Measures of primary outcomes

Human SD experiences included social isolation (n = 4), negative childhood experiences (n = 3), peer victimisation (n = 2), discrimination (n = 2), and chronic interpersonal stress (n = 2). The most frequently investigated markers of inflammation were serum IL-6 (n = 8) and CRP (n = 8), while others investigated additional markers including fibrinogen (n = 2) and inflammatory transcription factors such as nuclear factor kappa B (n = 1). There was a total of 21 investigated inflammatory markers. Depressive symptomology was the predominant mental health outcome (n = 10) assessed using various tools. Other mental health outcomes included PTSD (n = 2), internalising/externalising problems (n = 2), anxiety (n = 1) and loneliness (n = 1).

### Summary of included studies

Table 1 summarises the 13 studies included in this review, grouped by the main type of social-defeat (SD) experience examined—social isolation, peer victimisation, early-life adversity or childhood maltreatment, and discrimination or chronic stress. Each study is described in terms of its design, sample characteristics, SD construct, inflammatory markers, mental-health outcomes, analytical approach and principal findings. Grouping by SD construct and related inflammatory and psychological outcomes improves clarity and comparability across studies. Consistent terminology, column alignment, and standardised abbreviations were applied to enhance readability and adherence to PRISMA 2020 reporting standards.

Although a substantial body of research links childhood maltreatment and inflammation to severe mental illnesses such as schizophrenia and depression, most of these studies did not explicitly define or operationalise SD and therefore did not meet the inclusion criteria for this review.

### Narrative synthesis of findings

#### Associations between social defeat, inflammation, and mental health

##### General overview of results

The studies included in this review initially suggest that individuals who experience SD-like stressors are independently at greater risk of depressive symptoms [73, 75–77] and inflammation [72, 73, 76–81]. However, four studies [75, 77, 79, 82] only examined pairwise associations between variables rather than investigating interconnected relationships among all three factors. Conversely, nine studies [71–74, 76, 78, 80, 81, 83] provided more integrated analyses, with six [72, 73, 76, 80, 81, 83] investigating potential mediatory effects of inflammation (n = 4) or mental health diagnosis (n = 2) upon these relationships.

##### Independent associations across unintegrated studies

Among studies not directly testing associations between SD, inflammation, and mental health, Brody et al. [77] and Hsiao et al. [75] demonstrated positive associations between SD experiences and depressive symptoms. While Brody et al. [77] and Ong and Williams (2019) found no significant links between inflammatory markers and mental health outcomes, both found associations between discrimination and increased inflammation via elevated CRP, while Hsiao et al. [75] found social isolation to be marginally associated with elevated IL-6 in men. Conversely, Miller, Rohleder and Cole (2009) did not find any associations between chronic interpersonal stress with CRP and IL-6, but did find increased expression of mRNA for proinflammatory molecules (NF-κB and GR-β) amongst those experiencing chronic interpersonal stress. Interestingly, this was concurrent with an increase in mRNA coding for IκB, an inhibitor of NF-κB’s inflammatory effects, which positively correlated with depression.

##### Emerging patterns interlinking social defeat, inflammation, and mental health

The remaining nine studies identified associations between SD, inflammation, and mental health, focusing on four distinct SD experiences: social isolation, peer victimisation, early life adversity, and chronic interpersonal/social stress.

**Social Isolation:** Three cross-sectional studies [74, 78, 83] examined relationships between social isolation, inflammation, and mental health outcomes. While Ahmadian et al. [83] and Hsiao et al. [75] found no overall association between social isolation and inflammatory markers, Ahmadian et al. [83] reported that this relationship differed by PTSD status. Specifically, socially isolated individuals without PTSD exhibited higher CRP concentrations than their non-isolated counterparts, whereas this pattern was absent among those with PTSD. These findings suggest that PTSD may attenuate inflammatory responses to social isolation, possibly due to chronic HPA-axis hyperactivation and immune desensitisation observed in PTSD populations. Thus, rather than a contradiction, these results indicate that PTSD status moderates the link between social isolation and inflammation.

Complementing these findings, Häfner et al.‘s [78] multivariate regression analysis between middle-aged adults experiencing social isolation and depressed mood found no significant associations with hs-CRP. Interestingly, males demonstrated highly significant associations between combinations of social isolation, depressed mood, and IL-6. Similar sex-specific differences were shown by Koyama et al. [74] while comparing the combined effects of social isolation with inflammation and subjective feelings of loneliness. More specifically, men experiencing both social isolation and loneliness exhibited a higher neutrophil-lymphocyte ratio (NLR), suggestive of a shift towards a more inflammatory phenotype [74].

**Peer Victimisation:** Two studies investigated associations between peer victimisation, inflammation, and mental health outcomes [72, 76]. Arana et al. [76] identified statistically significant positive associations between social victimisation, inflammatory markers (CRP and IL-6), and depressive symptoms, while Roberts, Francesconi and Flouri (2023) found victimisation-associated increases in IL-6 and CRP that correlated with emotional, conduct, and peer problems as well as hyperactivity in children aged 7-11 years. Path mediation analyses also identified significant partial mediatory effects of peer victimisation on peer problems via IL-6. However, no mediatory associations were found for externalising problems. Similarly, Arana et al. [76] found no evidence supporting the role of inflammation as a mediator between victimisation and mental health outcomes.

**Early Life Adversity:** Studies investigating early life adversity (ELA) examined immunological responses spanning both adaptive and innate branches of the immune system [71, 73, 80]. While Ota et al. [80] found that childhood adversity was associated with differential expression of immune-related genes (e.g., *NR3C1*, *SMAD4*), Bielas et al. [71] reported alterations in adaptive immune markers, including T-cell subsets, lymphocyte counts, and Recent Thymic Emigrants (RTEs), whereas Iob et al. [73] assessed innate immune activation using CRP as an acute-phase inflammatory marker. Although each set of findings indicates immune dysregulation following early adversity, they represent distinct biological processes and should not be interpreted as interchangeable. For instance, adaptive immune markers reflect long-term cellular immune function and thymic activity, while CRP and cytokines (e.g., IL-6, TNF-α) index systemic inflammation mediated by the innate immune system. In Iob et al. [73], causal-mediation analysis tested whether CRP statistically mediated the relationship between adverse-childhood-experience exposure and subsequent depressive symptoms; the non-significant indirect effect suggested that, although CRP elevations accompany ELA, they do not explain its link with depression. This implies that inflammatory activation may represent a correlate rather than a mechanistic mediator of depressive risk, highlighting the likelihood of other psychosocial or biological pathways. This was similarly the case for Ota et al. [80] where immune-related gene (e.g., *NR3C1*, *SMAD4*) expression neither mediated nor moderated links between adversity and internalising/externalising symptoms. Distinguishing these mechanisms clarifies that ELA may confer vulnerability through multiple immunological routes—chronic inflammatory priming on one hand and altered lymphocyte regeneration on the other. Future reviews would benefit from synthesising adaptive and innate responses separately to strengthen mechanistic coherence.

**Chronic Interpersonal/Social Stress:** Von Guttenberg, Gassen, and Slavich (2025) investigated associations between chronic interpersonal/social stress, transcriptional signature and depressive symptoms in adolescent females. They reported that neural threat-network connectivity during negative social evaluation predicted acute shifts in innate immune transcriptional pathways (e.g., interferon/HIF-1), an effect that was mediated by one’s depression risk in adolescent female.

##### Heterogeneity and sensitivity analyses between studies

While formal statistical tests for heterogeneity and sensitivity analyses are not possible for narrative reviews, the range of study designs, SD operationalisations, inflammatory markers, and mental health outcomes suggests high heterogeneity between studies. To identify potential outcome variances, results were stratified and descriptively compared based on key study characteristics.

##### Descriptive observations of cross-study characteristics

No obvious differences were observed when comparing and grouping studies by multiethnicity (<65% one ethnicity), sex distribution, sample size, or country of publication. Cross-sectional studies accounted for 55.6% of the investigations that integrated and found associations between SD, inflammatory markers, and mental health outcomes. Age-based inspection showed that all studies integrating all three variables with a mean study population age >25 years exclusively examined social isolation effects. Methodological heterogeneity precluded further analysis by specific tools and analytical approaches used across studies.

##### Descriptive observations of sex differences across studies

Four studies identified sex differences, illustrating certain SD-related inflammatory responses to predominantly affect males. Häfner et al. [78] found associations between social isolation, depressed mood, and increased IL-6 in men, while Koyama et al. [74] identified social isolation and loneliness as being associated with increased NLR in men also. Hsiao et al. [75] additionally observed marginally increased IL-6 in socially isolated men while Iob et al. [73] reported increased CRP in males between 12–18 following sexual abuse. These findings suggest a potential male-specific vulnerability to inflammation in response to SD experiences.

##### Descriptive observations of group differences between social defeat, inflammation, and mental health

Descriptive observations identified nuances of potential interrelationships across studies based on SD exposure, measured inflammatory markers, and mental health outcomes. The following analyses exclude Miller, Rohleder and Cole (2009), Brody et al. [77], Ong and Williams (2019) and Hsiao et al. [75] due to their lack of integrated variables, altering the dataset to nine studies.

**Social Defeat:** Analysis by SD typology revealed consistent associations of peer victimisation (n = 2) and early life adversity (n = 3) with increased inflammatory markers and adverse mental health outcomes. Social isolation studies (n = 3) also showed associations, albeit primarily across male populations.

**Inflammation:** The most frequently assessed markers were CRP (n = 6) and IL-6 (n = 4). Häfner et al. [78], Ahmadian et al. [83] and Koyama et al. [74] found no effect of SD on CRP levels, with all three notably investigating social isolation. Conversely, studies on bullying [73] and peer victimisation [76] indicated positive relationships between SD, CRP, and depressive symptoms. Interestingly, Ahmadian et al. [83] demonstrated associations between social isolation and increased CRP but only for non-PTSD populations. Loneliness was inversely associated with CRP in women after adjusting for social isolation [74] while increasing CRP appeared to correlate with a reduction in individual risk of hyperactivity [72].

Regarding IL-6, Häfner et al. [78] and Arana et al. [76] demonstrated associations between SD, elevated IL-6 and increased depressive symptoms. Roberts, Francesconi and Flouri (2023) additionally identified a partial mediatory effect of IL-6 between peer victimisation and childhood peer problems.

**Mental Health:** Depressive symptoms were the most frequently assessed outcome (n = 6/9), followed by PTSD symptoms (n = 2/9) and Internalising/Externalising symptoms (n = 2/9). Häfner et al. [78] and Arana et al. [76] demonstrated SD-associated increases of IL-6 and depressive symptomology in the context of SD while Arana et al. [76] and Iob et al. [73] demonstrated elevated levels of CRP. Furthermore, Bielas et al. [71] linked depression in maltreated youth with significant reductions in RTE cells, a lineage associated with adaptive immunity (Ao et al., 2022).

##### Descriptive observations of study quality on results

A supplementary analysis excluding high ROB studies and those lacking integrative analysis included seven studies [71–73, 78, 80, 81, 83]. While each study identified SD-inflammation-mental health associations, no clear thematic commonalities arose, which remained after further restricting this sample to include only high-quality studies. Examination of studies exclusively with a high ROB [76, 78] showed no consistent patterns. These observations highlight inconsistencies among investigations when categorised by quality and ROB ratings, indicating a lack of uniformity in research rigour across studies.

##### Mechanisms and theoretical frameworks

Ten studies provided frameworks explaining their findings.

##### HPA axis dysregulation and sympathetic activation

Miller, Rohleder and Cole (2009), Häfner et al. [78] and Ota et al. [80] propose SD’s activation of stress response systems may influence independent relationships between SD, inflammation, and mental health. More specifically, while Häfner et al. [78] suggest cytokine-induced glucocorticoid receptor resistance as a mediator between social isolation, inflammation, and mental health, Miller, Rohleder and Cole (2009) posit chronic interpersonal stress may lead to cortisol resistance, diminishing its anti-inflammatory effects, creating an environment conducive to increased inflammation. They further propose that changes in inflammatory markers secondary to interpersonal stress might be attributed to sympathetic activation, specifically through norepinephrine-induced increases in NF-κB binding activity.

##### Health behaviours and gene expression

Ong and Williams (2019), Koyama et al. [74], Hsiao et al. [75] and Ota et al. [80] comprise an alternative hypothesis, suggesting health behaviours characteristic of those who have experienced SD may affect inflammation. These behaviours include disrupted sleep patterns and reduced physical activity which may alter autonomic and neuroendocrine functioning secondary to chronic stress, promoting inflammation-related gene expression. Arana et al. [76] alternatively suggests that specific SD experiences may activate protective genetic mechanisms - such as dopamine D2 receptor gene expression - associated with lower inflammation, potentially serving as a biological defence against the pro-inflammatory effects of psychological distress. This idea is shared by von Guttenberg et al. [81] which adds that social stress may increase depression risk through altered neural threat responses that trigger upregulation of inflammatory gene expression, reflecting heightened biological sensitivity to social threat.

##### Alternative perspectives

Other notable hypotheses include Bielas et al.‘s [71] suggestion that experiences of childhood maltreatment may accelerate age-related reductions in RTEs within a pro-inflammatory milieu, proposing that social stress could accelerate immunosenescence, i.e. the decline of immune function [84]. Meanwhile, Ahmadian et al. [83] alternatively suggests that inflammation might be intrinsic to the pathogenesis of mental health conditions, such as PTSD, positing that individuals with PTSD may exhibit altered inflammatory profiles.

## Discussion

This review set out to examine the relationship between SD, inflammation, and mental health outcomes through systematic review and narrative synthesis. Our findings suggest that individuals subjected to SD demonstrate immunological changes generally indicative of heightened inflammation, which are concurrently associated with mental health outcomes. A diverse range of human SD experiences varyingly predict elevations of CRP, IL-6, transcriptional signatures (e.g. mRNA for IκB), and reduced RTE cells, each of which were found to be associated with negative mental health outcomes. However, associations between SD, inflammation, and mental health may primarily be driven by SD-mental health or SD-inflammation relationships, rather than direct interrelations between all three variables. This review builds upon animal literature by supporting associations between repeated SD and raised pro-inflammatory cytokines within humans [33]. Similarly, our findings align with the growing evidence in humans supporting the role of inflammation across various psychiatric disorders [19, 85] while also challenging simplistic notions proposing that SD influences mental health solely through formal inflammatory pathways. Moreover, this review adds that the mechanisms by which SD relates to inflammation are multifaceted, including through altered health behaviours, gene expression and stress response systems.

Available evidence suggests that inflammatory activation generally follows, rather than precedes, exposure to SD. Longitudinal human studies (e.g., Iob et al., [73]) indicate that early-life social stressors predict later elevations in CRP and IL-6 [73], while experimental and animal paradigms show rapid post-defeat cytokine increases. This temporal pattern supports a stress-reactive, rather than pre-existing, inflammatory response. Mechanistically, SD engages the HPA and sympathetic–adrenal systems, whose neuroendocrine outputs (glucocorticoids, catecholamines) activate pro-inflammatory transcriptional pathways such as NF-κB and AP-1, leading to cytokine release [78, 82]. Although inflammation most plausibly arises after SD exposure, some evidence suggests that pre-existing low-grade inflammatory states may increase vulnerability to social stress, reinforcing a potential bidirectional feedback loop between immune dysregulation and psychosocial adversity [17, 52]. Alternatively, von Guttenberg et al. [81] shows that threat-network connectivity during negative social evaluation in adolescents predicts the *direction and magnitude* of innate immune transcriptional responses, including interferon and hypoxia signalling, linking social-threat neurocircuitry to immune activation in humans.

Evidence from animal and human studies further suggests that inflammation may have originated as an adaptive short-term response to social threat. Acute cytokine elevations following SD are thought to prepare the organism for potential injury or infection by enhancing immune vigilance and energy redistribution [31, 33]. Recent preclinical work suggests that the S100a9 (calprotectin) system may buffer the inflammatory and behavioural consequences of repeated SD stress, indicating that intrinsic immune-regulatory pathways might modulate vulnerability to SD-related pathology [86]. However, when social stressors are chronic or repeated, these same pathways remain persistently activated, leading to glucocorticoid resistance and sustained inflammatory signalling [78, 82]. Thus, a mechanism that is evolutionarily protective in the short term can become maladaptive when continually engaged, contributing to the inflammatory burden observed in stress-related mental disorders.

From an evolutionary standpoint, inflammation’s centrality in the SD–mental-illness chain may reflect its origin as a social-defence adaptation. Transient immune activation following exclusion or subordination would have conferred survival benefits by preparing for potential wounding and infection. In contemporary environments, however, persistent activation of this ancestral defence—without physical resolution of the threat—yields chronic low-grade inflammation and behavioural changes such as fatigue, social withdrawal, and anhedonia. These once-adaptive responses thus become maladaptive under sustained psychosocial stress, helping explain the evolutionary persistence yet pathological consequences of inflammation in SD-related mental disorders [87, 88].

### Inconsistencies with previous literature

The predominant lack of direct inflammation-mental health relationships in our findings contrasts extant literature outlining associations of CRP and IL-6 with depression [19, 25]. Combined with the lack of an identified mediatory role of inflammation to depression in this review, this contradicts elements of the social transduction framework, which links social stress to depressive symptoms via inflammatory upregulation [51]. Consequently, inflammation might not be a universal pathway for mental disorders but rather one of several upstream risk factors and thereby potentially less applicable to those experiencing SD-related mental health outcomes. Consistent with this overall pattern, Ota et al. [80] observed adversity-related shifts in immune gene expression without evidence that these transcripts mediated or moderated links to psychopathology, reinforcing that inflammation may act as a correlate or upstream risk signal rather than a consistent mediator across contexts.

Moreover, despite prior SD research predominantly focusing on schizophrenia and psychosis, no studies in this review examined these outcomes. This may reflect the potential unintentional exclusion of eligible studies within this review due to semantic issues defining and therefore excluding neuropsychiatric comorbidities during screening. Alternatively, these findings may highlight potential gaps in our evidence base of SD-inflammation relationships in schizophrenia and psychosis within humans. This systematic review presents the first comprehensive examination of the associations between SD, inflammation, and mental health, providing translational insights into SD-related inflammation in humans [33–36]. By integrating evidence across various manifestations of SD experiences, inflammatory markers, and mental health outcomes, this review highlights SD as an ecologically valid construct across human populations. Finally, this review demonstrates methodological rigour through adherence to PRISMA guidelines and the use of recognised quality assessment tools.

### Limitations of the current review

This review has several limitations. First, a key limitation is the small number of studies (n = 13) meeting the inclusion criteria, which reflects the limited empirical research directly examining human SD, inflammation, and mental health to date. Although this restricts the ability to draw firm causal conclusions, it highlights a critical evidence gap and reinforces the need for coordinated, longitudinal, and mechanistic studies to advance this emerging field. Second, of the studies examined in this review, six studies (46%) were cross-sectional and only two studies received a low ROB rating. Nevertheless, the inclusion of diverse study designs allowed for an in-depth investigation of the research topic, while varying levels of ROB underscore the need for more robust longitudinal studies. Third, though the use of a standardised extraction form and adherence to PRISMA guidelines helped maintain consistency and minimise potential biases ultimately data extraction and analyses were conducted by a single reviewer, increasing susceptibility to biases and errors. Although methodological cross-checks were performed and a random subset was verified by a second team member, this process did not fully meet the PRISMA recommendation for dual independent data extraction and quality appraisal. This limitation is acknowledged, and future reviews should incorporate independent double-review procedures to enhance reliability. Fourth, only five studies (38%) examined pairwise associations, limiting the ability to draw conclusions about comprehensive SD-inflammation-mental health interrelationships. However, these studies still provide valuable insights into specific aspects of these relationships within the broader picture of SD, inflammation, and mental health. Fifth, retrospective changes of the exclusion criteria before full-text screening may have introduced selection biases. Nevertheless, considering the heterogeneity identified within this review, these changes likely enhanced the focus of the final analysis. However, as highlighted, the potential semantic exclusion of ‘neuropsychiatric disorders’ may have led to the unintended exclusion of eligible studies during screening, potentially limiting the comprehensiveness of the review and the breadth of its findings and conclusions.

### Resilience and protective mechanisms

While much research has focused on vulnerability pathways linking SD to inflammation and mental ill-health, emerging evidence highlights the role of resilience mechanisms in modulating these effects. Psychosocial resources such as perceived social support, emotion regulation, and cognitive reappraisal can attenuate stress-related inflammatory responses by dampening HPA-axis and sympathetic activation [52]. Complementing this, animal research highlights inflammatory-sensitive pathways of both risk and resilience to social stress, emphasising individual variability in immune responsiveness and behavioural adaptation [89]. Similarly, positive affect, secure attachment, and a sense of purpose have been associated with lower basal IL-6 and CRP levels among individuals exposed to chronic social stress [90, 91]. Biological buffering processes, including enhanced parasympathetic (vagal) tone and anti-inflammatory cytokine activity such as IL-10, may further promote adaptive recovery [92]. Evidence from a recent meta-analysis also indicates that individuals with histories of childhood maltreatment show reduced resilience in adulthood, supporting the notion that early adversity may compromise adaptive biological and psychological responses to later social stress [93]. Integrating these perspectives underscores that SD-related inflammation is not deterministic but shaped by the dynamic interplay between risk and resilience factors.

### Clinical implications

The clinical implications of this review are twofold. First, this review highlights the importance of addressing psychosocial adversities experienced by marginalised communities through identification of SD-mental health associations and broader knowledge of the social determinants of mental health [8]. Second, while this review provides preliminary non-causal evidence supporting interrelationships between SD, inflammation, and mental health, should inflammatory correlates be identified, they could feasibly serve as biomarkers of mental disorders in the future. Inflammatory correlate data - likely with a particular focus on CRP and IL-6 as identified within this review – could inform future machine learning models identifying distinct transdiagnostic signatures of mental disorders, similar to existing work [94]. The combination of both personal SD history and individual inflammatory profile data with pre-existing patient data could enhance mental disorder risk prediction and aid diagnosis, especially in borderline or atypical cases.

### Recommendations and future directions

Based on the findings of this review, primary research should prioritise longitudinal study designs with standardised measurements of inflammation and mental health outcomes. These may help to more accurately confirm the existence of relationships between human SD, inflammation, and mental health by identifying specific markers of interest that may be used within predictive models. This would additionally create a more homogenous sample set of studies from which future and potentially more specific systematic reviews may develop.

Moreover, this review has largely challenged inflammation’s role as a mediator of SD-related mental health outcomes, namely depression, prompting questions about alternative mediating factors. Indeed, while this review has identified several theoretical frameworks to understand these relationships, the field may benefit from future research specifically aiming to identify specific mediators, creating a nuanced holistic framework by which one may understand SD.

## Conclusion

This is the first systematic review to examine the complex interrelationships between real-world SD, inflammation, and mental health in humans. Our findings generally support the hypothesis that SD experiences are associated with increased pro-inflammatory markers and negative mental health outcomes, while challenging the notion of universal relationships between SD, inflammation, and mental health. However, future research must focus on larger longitudinal studies that standardise inflammatory and mental health measures while exploring alternative mediators beyond inflammation in the pursuit of a more comprehensive framework for understanding the SD-mental health outcomes. Regardless, our findings offer translational support for animal studies linking SD to raised inflammatory markers, aligning with the growing recognition of inflammation’s relevance in psychiatric disorders.

## Supplementary information

Supplementary Table S.1

Supplementary Table S.2

Supplementary Table S.3
