# Supplementary material for: Inflammation as a mediating pathway between social defeat and mental health in humans: A systematic review
Source: Transl Psychiatry. 2026 Apr 24;16:304. doi: 10.1038/s41398-026-03911-z (PMC13237030; doi:10.1038/s41398-026-03911-z)
Supplement: Supplementary file 1 — Supplementary Table S.1 [file 41398_2026_3911_MOESM1_ESM.docx]

Supplementary Table S.1: Systematic Review Search Protocol

Presented as per PRISMA-S guidelines (62).

| Section/Topic | Item # | Description |
| --- | --- | --- |
| Information Sources and Methods | | |
| Database name | 1 | MEDLINE, PsycInfo, and Embase. |
| Multi-database searching | 2 | Whilst it was possible to run MEDLINE, PsycInfo, and Embase strategies simultaneously as a multi-file search via Ovid, each database was searched independently using the same Boolean parameters. This allowed for easier identification and resolution of database-specific issues or discrepancies in search results. |
| Study registries | 3 | No trial registries were searched within this study as it was deemed that information available on these registries, such as study protocols and summaries, may not be directly relevant to this study’s research question and may therefore not significantly contribute to the synthesis of evidence. |
| Online resources and browsing | 4 | Grey literature was not searched within this trial due to time constraints and the challenges of navigating an unstructured body of evidence.  Generalised search engines were not used for supplementary results due to the lack of structured indexing of results. |
| Citation searching | 5 | Whilst reference lists of included articles were not screened due to time constraints, future research may benefit from this. |
| Contacts | 6 | Whilst ideally authors and experts would be contacted to seek additional data, this did not occur due to time constraints. However, future research may benefit from doing so. |
| Other methods | 7 | No additional information sources or search methods were used. |
| Search Strategies | | |
| Full search strategies | 8 | The following strategy was employed upon Ovid across MEDLINE, PsycINFO, and Embase:   1. Social defeat OR Minority groups OR Minorities OR Minoriti?ed OR Marginali?ed OR BAME OR BME OR excluded groups OR Social exclusion OR Social isolation OR Social rejection OR Racism OR Bullying OR Bully OR Social stress OR Ostraci* 2. Inflammation OR Inflammatory OR Inflammatory-marker OR Inflammatory-biomarker OR Biomarker OR Neuroinflammation OR Acute phase protein OR Adaptive immune system OR Complement cascade OR Cytokines OR Innate immune system OR Macrophage OR Microglia OR Monocyte OR Natural killer cell OR NKC OR T lymphocyte OR Thymic lymphocyte OR Th1 OR Th-1 OR Th2 OR Th-2 OR Th17 OR Th-17 OR Treg OR T-reg OR CD8+ T cell OR T helper OR T-helper OR B-lymphocyte OR Interleukin OR IL* OR TNF* OR Tumour necrosis factor OR Tumor necrosis factor OR TGF* OR Tumour growth factor OR Tumor growth factor OR IFN* OR Interferon OR CRP OR C reactive protein OR C-reactive-protein OR C-reactive protein OR Cytokine OR Chemokine OR Peripheral blood marker 3. Mental health OR Mental disorder OR Mental illness OR Mental stress OR Mental wellbeing OR Wellbeing OR Psychosis OR Depression OR Anxiety OR OCD OR Schizophrenia OR PTSD OR Psychiatric illness OR Psychiatric disorder OR psychiatric 4. 1 AND 2 AND 3   The following link details the exact search strategy used across databases: [Search Strategy](https://ovidsp.ovid.com/ovidweb.cgi?T=JS&NEWS=N&PAGE=main&SHAREDSEARCHID=1P6zYr7IR6CYfmO0efQRzKhLlav2hdDyR3BuFTVkT36BRz5IWxGgBkxnO0qIob6Gi) |
| Limits and restrictions | 9 | The search was limited to human studies, English language, and abstracts. |
| Search filters | 10 | No additional filters were used. |
| Prior work | 11 | Due to the unique focus of this study, search strategies from other literature reviews were unable to be reused or adapted. |
| Updates | 12 | No additional studies were included between the initial comprehensive literature search and the time of data analysis due to time constraints. |
| Dates of searches | 13 | Searches were conducted sequentially across databases to accommodate the varied availability of team members. These occurred on the 9^th^ of April 2024 for PsycInfo, the 10^th^ of April 2024 for MEDLINE, and the 1^st^ of May 2024 for Embase. |
| Peer Review | | |
| Peer review | 14 | The strategies were informally peer-reviewed by a senior researcher within the Birmingham Institute of Mental Health. |
| Managing Records | | |
| Total records | 15 | A total of 15,230 citations were retrieved across MEDLINE, PsycINFO, and Embase. |
| Deduplication | 16 | Covidence software (Veritas Health Innovation, 2020) initially automated the removal of 3538 duplicates. However, some duplicates surpassed this and were manually excluded during title/abstract screening phases. |
