# Supplementary material for: Inflammation as a mediating pathway between social defeat and mental health in humans: A systematic review
Source: Transl Psychiatry. 2026 Apr 24;16:304. doi: 10.1038/s41398-026-03911-z (PMC13237030; doi:10.1038/s41398-026-03911-z)
Supplement: Supplementary file 3 — Supplementary Table S.3 [file 41398_2026_3911_MOESM3_ESM.docx]

**Supplementary Table S.3: Completed PRISMA Checklist for this Systematic Review**

PRISMA Checklist (60).

| **Section and Topic** | **Item #** | **Checklist item** | **Location where item is reported** |
| --- | --- | --- | --- |
| **TITLE** | | |  |
| Title | 1 | Identify the report as a systematic review. | Reported. See first page and title |
| **ABSTRACT** | | |  |
| Abstract | 2 | See the PRISMA 2020 for Abstracts checklist. | Reported. See Abstract and PRISMA abstract checklist reported within the document of tables. |
| **INTRODUCTION** | | |  |
| Rationale | 3 | Describe the rationale for the review in the context of existing knowledge. | Reported. See introduction. |
| Objectives | 4 | Provide an explicit statement of the objective(s) or question(s) the review addresses. | Reported. See introduction. |
| **METHODS** | | |  |
| Eligibility criteria | 5 | Specify the inclusion and exclusion criteria for the review and how studies were grouped for the syntheses. | Reported. See methods. |
| Information sources | 6 | Specify all databases, registers, websites, organisations, reference lists and other sources searched or consulted to identify studies. Specify the date when each source was last searched or consulted. | Reported. See methods. |
| Search strategy | 7 | Present the full search strategies for all databases, registers and websites, including any filters and limits used. | Reported. See the document of tables, table 1. |
| Selection process | 8 | Specify the methods used to decide whether a study met the inclusion criteria of the review, including how many reviewers screened each record and each report retrieved, whether they worked independently, and if applicable, details of automation tools used in the process. | Reported. See methods. |
| Data collection process | 9 | Specify the methods used to collect data from reports, including how many reviewers collected data from each report, whether they worked independently, any processes for obtaining or confirming data from study investigators, and if applicable, details of automation tools used in the process. | Reported. See methods. |
| Data items | 10a | List and define all outcomes for which data were sought. Specify whether all results that were compatible with each outcome domain in each study were sought (e.g. for all measures, time points, analyses), and if not, the methods used to decide which results to collect. | Reported. See methods. |
|  | 10b | List and define all other variables for which data were sought (e.g. participant and intervention characteristics, funding sources). Describe any assumptions made about any missing or unclear information. | Reported. See methods. |
| Study risk of bias assessment | 11 | Specify the methods used to assess risk of bias in the included studies, including details of the tool(s) used, how many reviewers assessed each study and whether they worked independently, and if applicable, details of automation tools used in the process. | Reported. See methods. |
| Effect measures | 12 | Specify for each outcome the effect measure(s) (e.g. risk ratio, mean difference) used in the synthesis or presentation of results. | Not applicable. |
| Synthesis methods | 13a | Describe the processes used to decide which studies were eligible for each synthesis (e.g. tabulating the study intervention characteristics and comparing against the planned groups for each synthesis (item #5)). | Not applicable. Statistical outcomes are excluded since this systematic review employs a narrative synthesis approach. |
|  | 13b | Describe any methods required to prepare the data for presentation or synthesis, such as handling of missing summary statistics, or data conversions. | Not applicable. Statistical outcomes are excluded since this systematic review employs a narrative synthesis approach. |
|  | 13c | Describe any methods used to tabulate or visually display results of individual studies and syntheses. | Reported. See methods. |
|  | 13d | Describe any methods used to synthesize results and provide a rationale for the choice(s). If meta-analysis was performed, describe the model(s), method(s) to identify the presence and extent of statistical heterogeneity, and software package(s) used. | Reported. See methods. |
|  | 13e | Describe any methods used to explore possible causes of heterogeneity among study results (e.g. subgroup analysis, meta-regression). | Reported. See methods. |
|  | 13f | Describe any sensitivity analyses conducted to assess robustness of the synthesized results. | Reported. See methods. |
| Reporting bias assessment | 14 | Describe any methods used to assess risk of bias due to missing results in a synthesis (arising from reporting biases). | Not applicable. There were no missing results. |
| Certainty assessment | 15 | Describe any methods used to assess certainty (or confidence) in the body of evidence for an outcome. | Partially reported. See methods. |
| **RESULTS** | | |  |
| Study selection | 16a | Describe the results of the search and selection process, from the number of records identified in the search to the number of studies included in the review, ideally using a flow diagram. | Reported. See results and PRISMA diagram within the document of figures (Figure 2). |
|  | 16b | Cite studies that might appear to meet the inclusion criteria, but which were excluded, and explain why they were excluded. | Not reported. |
| Study characteristics | 17 | Cite each included study and present its characteristics. | Reported. See results and Table 4 within the document of tables. |
| Risk of bias in studies | 18 | Present assessments of risk of bias for each included study. | Reported. See results and Table 4 within the document of tables. |
| Results of individual studies | 19 | For all outcomes, present, for each study: (a) summary statistics for each group (where appropriate) and (b) an effect estimate and its precision (e.g. confidence/credible interval), ideally using structured tables or plots. | Not applicable. Statistical outcomes are excluded since this systematic review employs a narrative synthesis approach. |
| Results of syntheses | 20a | For each synthesis, briefly summarise the characteristics and risk of bias among contributing studies. | Reported. See results and Table 4 within the document of tables. |
|  | 20b | Present results of all statistical syntheses conducted. If meta-analysis was done, present for each the summary estimate and its precision (e.g. confidence/credible interval) and measures of statistical heterogeneity. If comparing groups, describe the direction of the effect. | Not applicable. Statistical outcomes are excluded since this systematic review employs a narrative synthesis approach. |
|  | 20c | Present results of all investigations of possible causes of heterogeneity among study results. | Descriptive observations of heterogeneity are reported. See results. |
|  | 20d | Present results of all sensitivity analyses conducted to assess the robustness of the synthesized results. | Descriptive observations and variances of ‘sensitivity analyses’ are reported. See results. |
| Reporting biases | 21 | Present assessments of risk of bias due to missing results (arising from reporting biases) for each synthesis assessed. | Not applicable. No missing results. |
| Certainty of evidence | 22 | Present assessments of certainty (or confidence) in the body of evidence for each outcome assessed. | Not reported. |
| **DISCUSSION** | | |  |
| Discussion | 23a | Provide a general interpretation of the results in the context of other evidence. | Reported. See discussion. |
|  | 23b | Discuss any limitations of the evidence included in the review. | Reported. See discussion. |
|  | 23c | Discuss any limitations of the review processes used. | Reported. See discussion. |
|  | 23d | Discuss implications of the results for practice, policy, and future research. | Reported. See discussion. |
| **OTHER INFORMATION** | | |  |
| Registration and protocol | 24a | Provide registration information for the review, including register name and registration number, or state that the review was not registered. | Reported. See methods. |
|  | 24b | Indicate where the review protocol can be accessed, or state that a protocol was not prepared. | Not reported. |
|  | 24c | Describe and explain any amendments to information provided at registration or in the protocol. | Not applicable. |
| Support | 25 | Describe sources of financial or non-financial support for the review, and the role of the funders or sponsors in the review. | Not applicable – there were no funders or sponsors. |
| Competing interests | 26 | Declare any competing interests of review authors. | Not applicable – no competing interests to declare. |
| Availability of data, code and other materials | 27 | Report which of the following are publicly available and where they can be found: template data collection forms; data extracted from included studies; data used for all analyses; analytic code; any other materials used in the review. | Full data extraction is available by request; access to included studies may be found within the references. |
